# Gene Expression Profiling of the Shoot Meristematic Tissues in Woodland Strawberry *Fragaria vesca*

**DOI:** 10.3389/fpls.2019.01624

**Published:** 2019-12-13

**Authors:** Yongping Li, Jia Feng, Laichao Cheng, Cheng Dai, Qi Gao, Zhongchi Liu, Chunying Kang

**Affiliations:** ^1^Key Laboratory of Horticultural Plant Biology (Ministry of Education), College of Horticulture and Forestry Sciences, Huazhong Agricultural University, Wuhan, China; ^2^National Key Laboratory of Crop Genetic Improvement, College of Plant Science and Technology, Huazhong Agricultural University, Wuhan, China; ^3^Department of Cell Biology and Molecular Genetics, University of Maryland, College Park, College Park, MD, United States

**Keywords:** shoot apical meristem, flower meristem, receptacle meristem, RNA-seq, transcription factor, hormone, *FvWUS1*, F. vesca

## Abstract

*Fragaria vesca*, a wild diploid strawberry, has recently emerged as a model for the cultivated strawberry and other members of the Rosaceae. Differentiation and maintenance of meristems largely determines plant architecture, flower development and ultimately fruit yield. However, in strawberry, our knowledge of molecular regulation of meristems in different developmental context is limited. In this study, we hand dissected three types of tissues than contain meristematic tissues corresponding to shoot apical meristem (SAM), flower meristem (FM), and receptacle meristem (REM), in *F. vesca* for RNA-seq analyses. A total of 3,009 differentially expressed genes (DEGs) were identified through pairwise comparisons. These DEGs were grouped into nine clusters with dynamic and distinct expression patterns. In these nine clusters, 336 transcription factor genes belong to 46 families were identified; some of which were significantly enriched in FM and REM such as the MADS-box family or in REM such as the B3 family. We found conserved and distinctive expression patterns of totally 149 genes whose homologs regulate flowering time or SAM, leaf, and flower development in other plant species. In addition to the ABCE genes in flower development, new MADS box genes were identified to exhibit differential expression in these different tissues. Additionally, the cytokinin and auxin pathway genes also exhibited distinct expression patterns. The Arabidopsis homeobox gene *WUSCHEL* (*WUS*), essential for stem cell maintenance, is expressed in organizing center of meristems. The *F. vesca* homolog *FvWUS1* exhibited a broader expression domain in young strawberry flowers than its Arabidopsis counterpart. Altogether, this work provides a valuable data resource for dissecting gene regulatory networks operating in different meristematic tissues in strawberry.

## Introduction

Strawberries are herbaceous perennials with unique flower and fruit and distinct plant architectures, such as numerous individual carpels grown on the receptacle of flowers and accessory fruit (Hollender et al., 2012). The cultivated strawberry (*Fragaria × ananassa*, octoploid) is an important fruit crop worldwide. Together with the cultivated strawberry, there are about 25 recognized *Fragaria* species in the world, with different ploidies from diploid to decaploid (Staudt, 2009). *F. vesca* is a diploid progenitor species of *F. × ananassa* (Edger et al., 2019) and frequently used as a model for cultivated strawberry which is octoploid and a hybrid. One advantage of *F. vesca* is its high quality genome (Shulaev et al., 2011; Edger et al., 2018). Previously, we profiled the transcriptomes of flower and fruit tissues at different developmental stages in *F. vesca* (Kang et al., 2013; Hollender et al., 2014). However, the transcriptomes of some important strawberry tissues are still lacking.

Meristems contain stem cells of plants. The shoot apical meristem (SAM) is located on the stem tip, which is further divided into three zones, namely the central zone (CZ), the organizing center (OC), and the peripheral zone (PZ). In strawberry, during vegetative phase, SAM gives rise to new lateral leaf primordia and new lateral branches (branch crown); upon floral induction, SAM becomes inflorescence meristem, which terminates in a floral meristem (FM). As the strawberry inflorescence belongs to dichasial cyme (the primary flower is determinate, and secondary flowers emerge from the base of the peduncle) (Hollender et al., 2012), it is difficult to distinguish floral meristem from inflorescence meristem. In strawberry flower meristem (FM) gives rise to the four whorls of flower organs. Their carpels are not fused and numerous individual carpels emerged from the receptacle sequentially from the base to apex. Thus, the receptacle of a young flower bud maintains its meristematic activity for an extended period of time to continuously give rise to new carpel primordia. Hence, the receptacle is like an FM except that it gives rise to carpels only. For this reason, the developing receptacle is designated as REceptacle Meristem (REM) in this study. Characterization of the REM transcriptome would unveil the underlying regulatory network that specifies its unique development, and facilitates comparative studies with plant species, whose flowers do not have an enlarged receptacle.

Much of our knowledge on the molecular mechanisms underlying SAM and FM development and floral organ identity determination is based on studies of Arabidopsis. The SAM maintenance is regulated by the *WUS*/*CLV* signaling module (Schoof et al., 2000; Braybrook and Kuhlemeier, 2010). *WUSCHEL* (*WUS*) is a transcription factor expressed in the OC and promotes stem cell fate (Laux et al., 1996; Mayer et al., 1998). *CLAVATA3* (*CLV3*) encodes a small peptide and is a marker gene of the CZ (Fletcher et al., 1999). The WUS protein could move to the CZ to promote the expression of *CLV3*, while *CLV3* and its receptors together inhibit expression of *WUS* (Brand et al., 2000; Schoof et al., 2000; Yadav et al., 2011; Daum et al., 2014), thus forming a negative feedback loop to maintain the proper size of stem cell population. The *WUS/CLV* signaling module functions in both SAM and FM to maintain their meristem identity. However, FM eventually terminates after four whorls of floral organs are formed. During flower development, four classes of genes (A, B, C, and E) interact to coordinately specify four floral-organ types (Coen and Meyerowitz, 1991; Krizek and Fletcher, 2005; Causier et al., 2010). Specifically, A (*APETALA1*/*AP1*, *AP2*) and E genes (*SEPALLATA*/*SEP* and *SEP-like*) together specify sepal identity; A, B (*AP3*, *PISTILLATA*/*PI*), and E genes specify petal identity; B, C (*AGAMOUS*/*AG*), and E genes specify stamen identity; C and E genes specify carpel identity. Most of the ABCE genes belong to the MADS-box gene family except *AP2*, which belongs to AP2 family of transcription factors (Parenicova et al., 2003; Arora et al., 2007; Smaczniak et al., 2012).

Thus far, no transcriptome is available for the SAM in strawberry. Previously, we analyzed the transcriptomes of Flower_1–4 (floral stages 1–4) and Receptacle_6–7 (floral stages 6–7) (Hollender et al., 2014), corresponding to FM and REM, respectively. Tissue-specific gene clusters were obtained for the two tissues. However, these tissues were collected by laser capture microdissection (LCM). We found that a lower percentage of raw reads were aligned to the coding sequences for the LCM libraries, that is, 30–40% for LCM and 60–70% for hand-dissected (Hollender et al., 2014). Since LCM and hand-dissection employ very different methods in tissue fixation, sampling, and library construction, a large number of differentially expressed genes are likely caused by technical differences. This is supported by the detection of different expression profiles between LCM and hand-dissection generated from the same tissue (Hollender et al., 2014). Thus, new transcriptomic data using hand-dissected tissues including FM and REM are desirable for comparisons with previously generated data using hand dissection.

In this work, Illumina RNA-seq is utilized to profile hand dissected SAM, FM, and REM tissues to gain insights into genome-wide gene expression dynamics in SAM, FM, and REM of *F. vesca*. The differentially expressed genes were divided into nine gene clusters with distinct expression patterns. A large number of well-known transcription factors and hormone genes were identified to be highly or specifically expressed in each meristem. One notable finding is that *FvWUS1*, a homolog of the stem cell maintenance gene *WUS* in Arabidopsis, is more abundantly expressed in the FM and REM than SAM, which was validated by the *FvWUS1pro::GUS* reporter in transgenic *F. vesca* lines as well as RNA *in situ* hybridization in *F. vesca*. This expression pattern of *WUS* in *F. vesca* is distinctly different from that in *Arabidopsis*. Together, our results provide a valuable resource for future functional dissection of key genes regulating plant architectures and flower development in strawberry.

## Materials and Methods

### Plant Materials and Tissue Isolation

**Figure 1:**
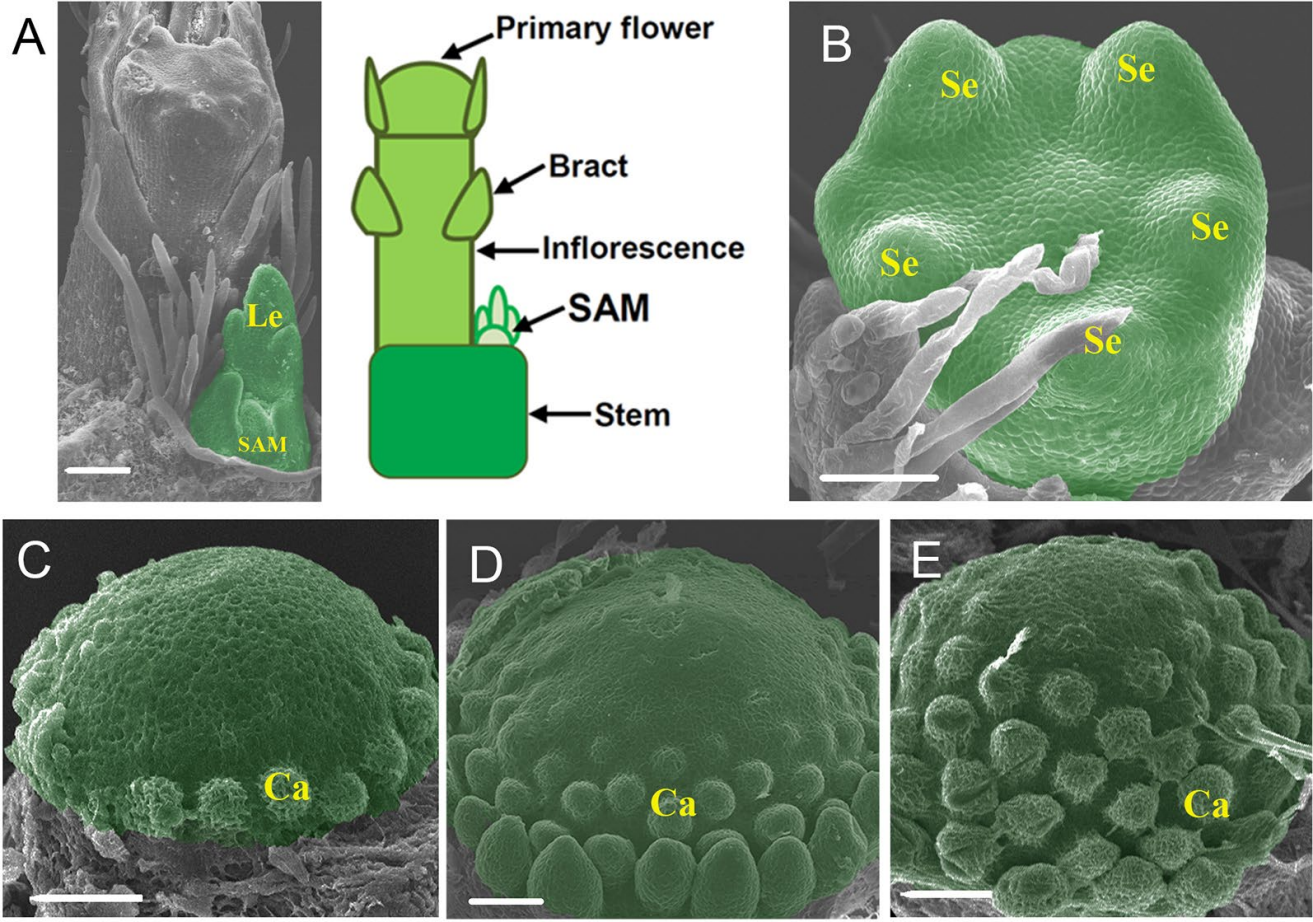
Morphology of the three meristems used for transcriptome analysis. **(A)** SEM image and diagram showing the tip of a strawberry (*F. vesca*) shoot with an inflorescence emerging. **(B)** SEM image of stage 3 flower. The entire flowers were collected as the FM (flower meristem). **(C**–**E)** SEM images of the receptacles at stages 5–7, respectively. Sepals, petals, and anthers were removed to expose the receptacles. These receptacles were pooled together as the REM (receptacle meristem). Le, leaf; SAM, shoot apical meristem; Se, sepal; Ca, carpel. The SAM, FM, and REM are false colored green, respectively. Scale Bars = 50 μm.

The tissues for RNA-seq were dissected manually from YW5AF7, an inbred line of the diploid *F. vesca* day-neutral variety Yellow Wonder (Slovin et al., 2009), using tweezers under a dissecting microscope. Plants used for tissue isolation were grown in substrate under natural conditions in plastic-covered tunnels in June and July at the Huazhong Agricultural University, when they were flowering. The *F. vesca* transgenic plants were grown in a growth room under a light intensity of 100 µmol m^−2^ s^−1^ with a 16/8 h light/dark photoperiod at 25°C. The samples SAM, FM, and REM were designated as described in **Figure 1**.

### RNA Extraction and Illumina RNA Sequencing

The tissues were dissected and frozen immediately in liquid N2, and then stored at −80 °C until use. Total RNAs were extracted with the RNeasy Plant Mini Kit (Qiagen, Cat# 74903). Three biological replicates for each tissue type were prepared. Approximately 1 µg of total RNAs per sample was sent to Beijing Genomics Institute (BGI, Wuhan, China) for strand specific library construction and sequencing on Illumina HiSeq2500. In total, 292 million 125 bp paired-end reads were generated. This dataset was deposited at the Sequence Read Archive (SRA) at NCBI (http://www.ncbi.nlm.nih.gov/sra) under the accession number SRP115444.

### Gene Expression Analysis

The first 12 nt of each read was trimmed off using fastx_trim in the FASTX-Toolkit (http://hannonlab.cshl.edu/fastx_toolkit/). The trimmed reads were mapped against the *F. vesca* V4 genome with the annotation v4.0.a2 using the program STAR in a 2-pass mode (Dobin et al., 2013; Edger et al., 2018; Li et al., 2019). Only the uniquely mapped reads were retained for further analysis. The aligned reads for each gene were summarized by the program featureCounts (Liao et al., 2014), and TPM (Transcripts Per Million) was used to represent the expression level. The multi-dimensional scaling (MDS) analysis was performed using the log2 transformed TPM of all the expressed genes for each sample with the Euclidean Distance statistical method. Pairwise comparisons were carried out using the R package DESeq2 (Love et al., 2014) to identify the differentially expressed genes (fold change >2 and padj <0.05). All the differentially expressed genes were subjected to K-means clustering with the Euclidean Distance metric in MeV4.8.1 (Saeed et al., 2003). The gene expression plot of each cluster was made using the R package ggplot2.

### WGCNA Network Analysis

Gene coexpression networks were constructed using the WGCNA (v1.68) package in R. A total of 97 Illumina RNA-seq libraries generated from 46 tissue types in *F. vesca* were downloaded to construct the gene coexpression network (Kang et al., 2013; Hollender et al., 2014; Toljamo et al., 2016; Hawkins et al., 2017; Li et al., 2018). A total of 26,192 genes with TPM >2 in at least one of the tissues were used for the WGCNA signed coexpression network analysis (Langfelder and Horvath, 2008), and the average TPM of the three biological replicates was used as the input. Gene modules were obtained using the automatic network construction function blockwiseModules with default settings, except power = 16, minModuleSize = 30, and mergeCutHeight = 0.25. The eigengene value was calculated for each module and used to test the association with each tissue type. The total connectivity and intramodular connectivity (function softConnectivity), kME (for modular membership), and kME-*P* value were calculated for all the genes, which were clustered into 31 modules. Gene network was visualized using Cytoscape_v3.7.1.

### Identification and Phylogenetic Analysis of the MADS-Box Genes in *F. vesca*

To globally identify the MADS-box genes in *F. vesca*, the SRF-TF domain (PF00319) was retrieved from the Pfam 27 database (Finn et al., 2013), and the protein sequences were obtained from the V4 genome according to the v4.0.a2 annotation (Edger et al., 2018; Li et al., 2019). The Hidden Markov Model-based HMMER program (version h3.1b2) was used to identify the putative MADS-box proteins with default settings. The full-length protein sequences of the Arabidopsis and strawberry MADS-box genes were aligned by using ClustalW. The unrooted phylogenetic tree was constructed with the MEGA 7 software (Kumar et al., 2016) with the Maximum Likelihood statistical method and bootstrap analysis (1,000 replicates).

### Scanning Electron Microscope

Samples were fixed in 2.5% glutaraldehyde at 4°C overnight, treated sequentially with 30%, 50%, 70%, 90% and 100% ethanol for 10 min each, transferred to isoamyl acetate for 20 min, critical point dried, coated with gold for 30 s, and photographed under a scanning electron microscope (JSM-6390LV).

### Plasmid Construction

To make the construct *FvWUS1pro::GUS*, the promoter of *FvWUS1* (FvH4_3g04400/gene30464) with a length of 2,076 bp upstream of the translational start codon was isolated by PCR amplification from the genomic DNA of YW5AF7 using the forward primer (5’- AGCGCTGAAGCTTGGCTGCAGAGTCCAAGATCTCTC​ACTTGC​AAG-3’) and the reverse primer (5’-GGACT​GACCAC​CCGGGGATCCTGGTGATGGGCTGAGAGAATGAGA-3’). The fragment was inserted into the binary vector DX2181G (Ye et al., 2012) digested by the PstI/BamHI restriction enzymes using the Gibson cloning method.

### Strawberry Transformation

*Agrobacterium*-mediated strawberry transformation was performed as previously described (Feng et al., 2019). Briefly, leaf strips of the *F. vesca* variety Hawaii 4 were used as explants, and positive transgenic calli and regenerated plants were screened using 4 mg l^−1^ hygromycin during transformation.

### Gus Staining

The SAM and flower buds at early developmental stages were dissected under a stereomicroscope from five independent T0 transgenic plants. The tissues were stored in cold 90% acetone during dissection and then kept at room temperature for 20 min. Next, acetone was removed and GUS staining solution (1 mM X-glucuronic acid, 0.1 mM EDTA, 0.1% Triton X-100, and 10 mM potassium ferri/ferrocyanide in 100 mM phosphate buffer, pH 7.0) was added to submerge all the materials. After 30 min of vacuum, tissues were incubated overnight at 37°C. The samples were then mounted on clearing solution (chloral hydrate: glycerol: H2O: 8:1:1) for 5 h and observed under differential interference contrast (DIC) optics using a Zeiss Axioscope A1 microscope with a ×0.5 optical adapter. The images were captured and exported using ZEN2.3.

### RNA *In Situ* Hybridization

The SAM and young flower buds of the *F. vesca* variety YW were fixed in the formaldehyde-acetic acid-ethanol fixative solution and stored at 4°C until use. Floral development stages were designated as previously described (Hollender et al., 2012). For the antisense probe, a 163 bp fragment of *FvWUS1* (21–183 bp in the coding sequence) was synthesized and inserted into the pSPT18 vector. The antisense probe was synthesized using the forward primer 5’-CGTTTCACAACTCTCACATGC-3’ and the reverse primer 5’-CATTGGTGATGGGCTGAGA-3’. The M13 fragment was used as the control probe. Synthesis of DIG-labeled RNA probes *via in vitro* transcription was carried out using the DIG RNA Labeling Kit (SP6/T7) (Roche, Cat# 11175025910). The hybridization signals were detected using the DIG Nucleic Acid Detection Kit (Roche, Cat# 11175041910). Slides were developed in a dark humid container for 24 h and development was stopped and stored with 1×TE. The images were captured using a Zeiss Axioscope A1 microscope with a ×0.5 optical adapter.

## Results and Discussion

### Morphology of the Three Shoot Meristem Tissues and RNA-Seq Analysis in *F. vesca*

The shoot apical meristem (SAM) of strawberry is deeply buried by young leaves and surrounded by a young leaf primordium. When flowering, an inflorescence is initiated that occupies a large space on the stem tip and pushes the SAM aside (**Figure 1A**). In this work, the sample SAM consisted of the typical SAM as well as a subtending leaf primordium and a short stem underneath (**Figure 1A**). The flower development in *F. vesca* was divided into 13 stages (Hollender et al., 2012). The sample FM consisted of flower primordia at stages 1 to 4. At stages 1 and 2, the flower primordia look like a dome. At stage 3, five sepal primordia appear (**Figure 1B**). At stage 4, the flower center is flat, petal primordia start to form, and short trichomes appear on sepals. REM consists of developing receptacles with the carpel primordia emerging from the base to the apex in a spiral pattern at floral stages 5 to 7 (**Figures 1C–E**). Approximately 70–100 shoot apexes or flower buds were collected by hand dissection to give sufficient quantities of total RNAs for each biological replicate.

**Figure 2:**
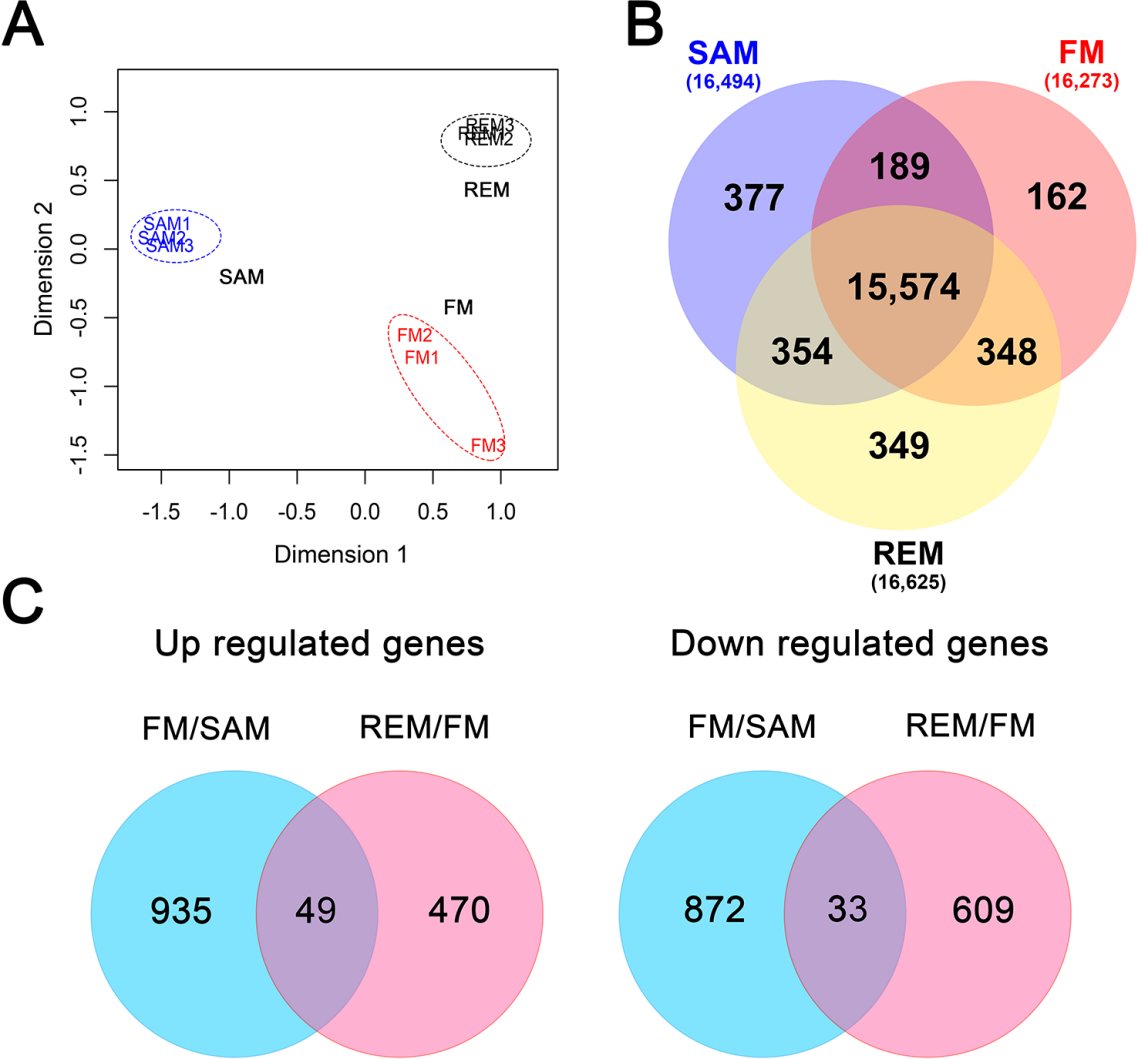
Global analysis of the meristem transcriptomes. **(A)** MDS plot showing the overall relationship of the three tissue types with three biological replicates. SAM, blue; FM, red; REM, black. Log2-transformed TPM was used with the Euclidean Distance method to measure the pairwise similarity. **(B)** Venn diagram showing the unique and common genes (TPM > 2) in each tissue. **(C)** Venn diagram showing the up-regulated and down-regulated genes in the pairwise comparisons (fold change > 2, padj < 0.05).

RNA-seq was carried out for a total of nine samples (three biological replicates for each meristem type) using the Illumina HiSeq-2500 platform (paired-end, 125bp). The number of raw reads ranged from 29.7 to 34.5 million for each sample, totaling 292.4 million, and an average of 87.7% of the raw reads were mapped to the FvH4 genome with the annotation ver4.0.a2 (**Supplementary Table S1**) (Li et al., 2019). Genes with a TPM (Transcripts Per Million) value lower than 2 were considered “lowly expressed” and removed from downstream analysis. As a result, 15,106–16,694 genes were expressed among the 34,007 annotated protein-coding genes in each transcriptome. The global relative relationship among the nine samples was examined by multi-dimensional scaling (MDS) plot analysis. In the MDS plot, the three tissue types were separated from each other (**Figure 2A**), indicating that they are quite distinct. Moreover, the three biological replicates for each tissue were grouped together, suggesting uniform sampling. A Venn diagram was used to reveal the uniquely or commonly expressed genes among the three tissues (**Figure 2B**). In total, 15,574 genes were commonly expressed, while only 162 genes (FM) to 377 genes (SAM) were specifically expressed in one tissue.

### Differentially Expressed Genes Accompanying Meristem Development

To identify differentially expressed genes (DEGs), pairwise comparisons were carried out between SAM and FM and between FM and REM, respectively, by using the R package DESeq2 (Love et al., 2014). From SAM to FM, 984 genes were significantly up-regulated in FM, while 905 genes were down-regulated in FM (fold change >2, padj ≤0.05) (**Figure 2C**). In the list of DEGs (**Supplementary Data 1**), the flower meristem identity genes *FvAPETALA1* (*FvAP1*, FvH4_4g29600) and *FvLEAFYa* (*FvLFYa*, FvH4_5g09660) as well as other well-known flower development genes were greatly up-regulated in FM. Gene ontology (GO) analysis revealed that the GO terms of the biosynthetic and metabolic processes were greatly enriched among the up-regulated genes in the FM compared to the SAM (**Supplementary Data 1**). From FM to REM, 519 and 642 genes were respectively up- and down-regulated in REM (fold change >2, padj ≤0.05) (**Figure 2C**, **Supplementary Data 1**). In this comparison, genes involved in the immune responses and transports were significantly enriched in the up-regulated genes. Intriguingly, 49 genes were continuously up-regulated at the two pairwise comparisons, some of which are the homologs of well-studied genes in other plant species, such as the floral meristem identity gene *FvLFYa* and flower development genes *FvAGAMOUS* (*FvAG*, FvH4_3g06720, C gene) and *FvSEPALLATA3* (*FvSEP3*, FvH4_4g23530, E gene) (Mouhu et al., 2013; Hollender et al., 2014). In addition, 33 genes were continuously down-regulated at the two pairwise comparisons, such as FvH4_7g28740 coding for a SOC1-like MADS box transcription factor, the homolog of *AGAMOUS-LIKE 42* that promotes floral transition in Arabidopsis (Dorca-Fornell et al., 2011), FvH4_2g21310 and FvH4_3g11120, the homologs of *TAWAWA1* regulating the meristem phase change in rice inflorescence (Yoshida et al., 2013).

### More DEGs Were Identified Between FM and REM Than the LCM Samples

Previously, we analyzed the transcriptomes of Flower_1–4 and Receptacle_6–7, corresponding to the FM and REM, respectively (Hollender et al., 2014). We wanted to see how the new data is related to the previous LCM data. First, we analyzed the overall relatedness of transcriptomes of the four transcriptome datasets. The MDS plot revealed that Flower_1–4 and Receptacle_6–7 are distantly separated from the three meristem samples generated in this study (**Supplementary Figure S1A**). Next, we identified the DEGs between Flower_1–4 and Receptacle_6–7 using the same analysis pipeline as described above for FM and REM. Only 200 DEGs were identified between Flower_1–4 and Receptacle_6–7 (**Supplementary Data 2**), in a sharp contrast to the 1,161 DEGs between FM and REM. Moreover, 54 out of the 131 up-regulated genes and 26 out of the 69 down-regulated genes were not included in the DEGs between FM and REM (**Supplementary Figure S1B**). Overall, the new transcriptomes generated in this study revealed a higher number of DEGs compared to the previous LCM data. The analyses also suggest that the techniques employed can significantly affect the RNA-seq results.

### Dynamic Expression of DEGs During Meristem Development

**Figure 3:**
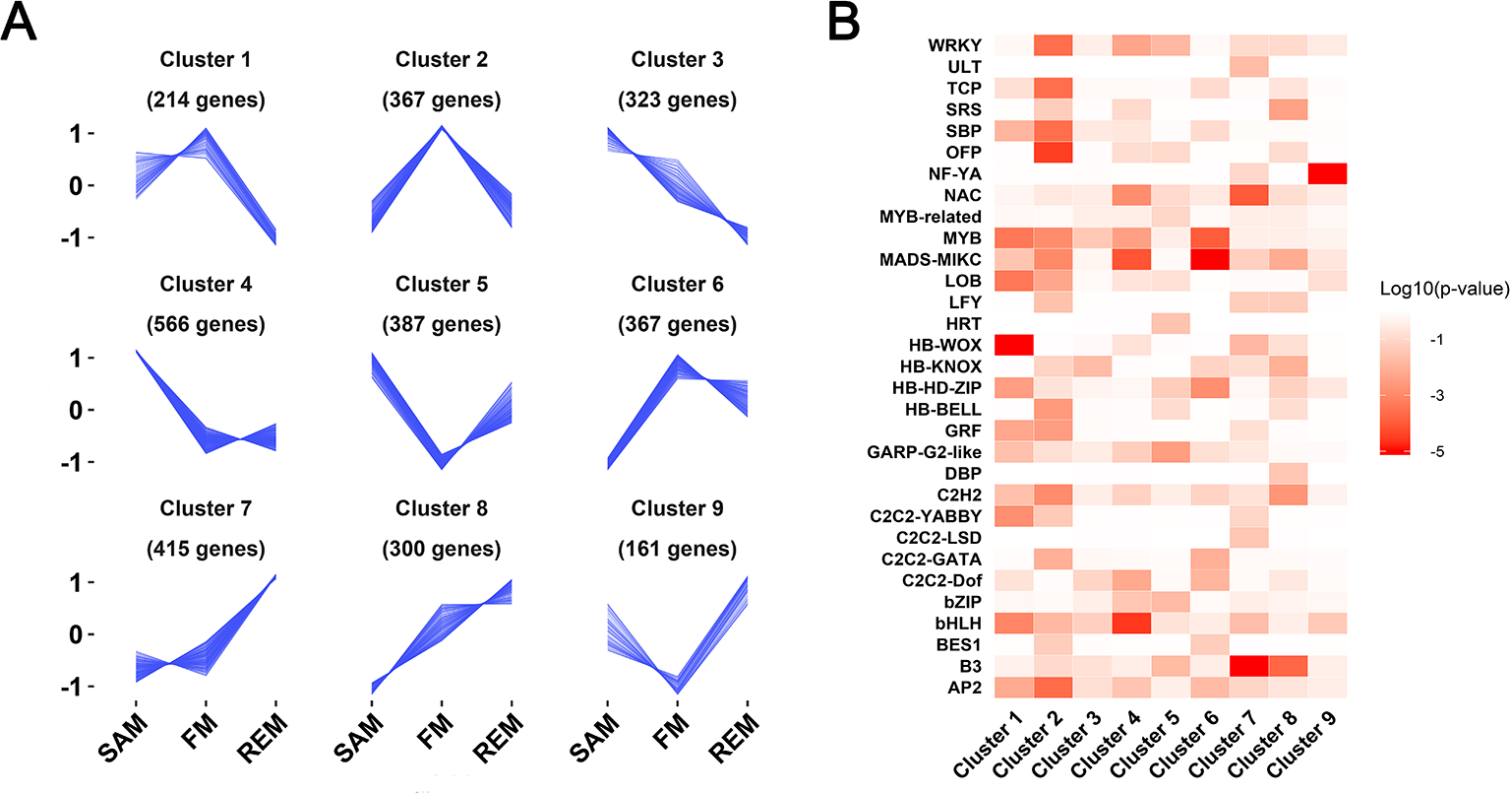
Nine gene clusters with distinct expression patterns. **(A)** Line plot showing the expression trends of nine gene clusters containing a total of 3,009 differentially expressed genes. Y-axis represents the normalized z-score obtained from the average TPM of three biological replicates. The number of genes in each cluster is indicated. **(B)** Heatmap showing the enriched transcription factor families in each of the nine gene clusters. Enrichment is indicated by the log10 transformed *P* values.

Combined the three pairwise comparisons (SAM and FM, FM and REM, SAM and REM), a total of 3,009 genes were found to be differentially expressed (fold change >2, padj ≤0.05). These genes were assigned into nine gene clusters with dynamic and distinct expression patterns using the k-means clustering algorithm (**Supplementary Data 3**). Of these, cluster 4, cluster 2, and cluster 7 contained genes predominantly expressed in the SAM, FM, and REM, respectively (**Figure 3A**). Cluster 4 contained the greatest number of genes, indicating the uniqueness or higher complexity of SAM. Expression of the cluster 8 genes was gradually increased from SAM to REM. In contrast, cluster 3 genes exhibited an opposing expression trend. In addition, cluster 1 genes were abundantly expressed in both SAM and FM. Cluster 6 genes were abundantly expressed in both FM and REM. Unexpectedly, cluster 5 and 9 genes were abundantly expressed in both SAM and REM. The genes in the nine clusters were also subjected to the GO analysis, respectively (**Supplementary Data 3**). Four out of the nine clusters (clusters 2, 5, 6, and 7) were found to possess enriched GO terms in the ‘Biological Process’ category.

Transcription factor genes play essential roles in the regulatory network of meristem maintenance and differentiation (Chen et al., 2018; Tian et al., 2019). Among the 3,009 DEGs that were grouped into nine clusters, 336 genes encode transcription factors belonging to 46 families (**Supplementary Data 3**). The enriched transcription factor families in each cluster were shown by a heatmap representing the log10 transformed probabilities (**Figure 3B**). Several clusters possess enriched transcription factor families. For instance, there are four HB-WOX family genes in cluster 1, which are highly expressed in the SAM and FM, but significantly reduced in the REM. Cluster 6 (lowly expressed in the SAM but highly expressed in FM and REM) is enriched with several flower development genes of the MADS-box family, including three B genes (*FvAP3*, FvH4_1g12260; *FvPIa*, FvH4_2g27860; *FvPIb*, FvH4_2g27870) and two E genes (*FvSEP4*, FvH4_5g13510; *FvSEP1-like*, FvH4_4g29610). Intriguingly, the B3 family genes (Swaminathan et al., 2008) are preferentially expressed in the REM (cluster 7), which may be involved in receptacle or carpel development. The NF-YA family, encoding a subunit in the heterotrimeric NF-Y transcription factor complex, is enriched in cluster 9, whose functions in the receptacle or carpel development need further investigations.

### Examination of Genes in Flowering Time Regulation

**Figure 4:**
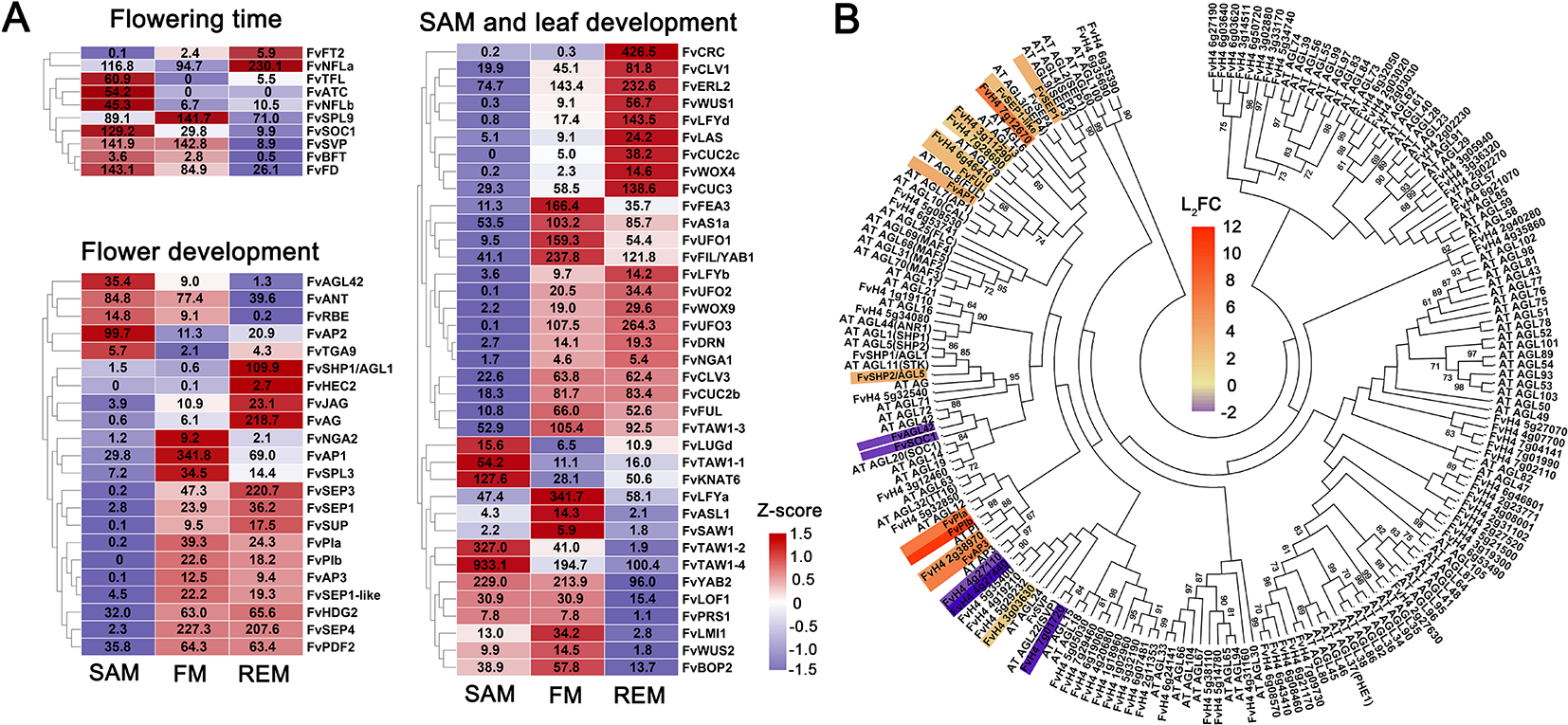
DEGs with known homologs and the MADS-box genes. **(A)** Heatmap showing the expression patterns of differentially expressed genes regulating the flowering time and development of SAM, leaf, and flower. Z-score obtained from averaged TPM of three biological replicates was used. The numbers in the heatmap indicate the TPM value of each gene in the tissues. **(B)** Phylogenetic tree showing all the MADS-box genes in *F. vesca* and Arabidopsis. The tree was generated by the Maximum Likelihood algorithm and bootstrap analysis (1,000 replicates) using MEGA 7. The differentially expressed MADS-box genes between the SAM and FM in our data were indicated with a scaled color (L_2_FC, log2 fold change). Red indicates up-regulation, and blue indicates down-regulation in the FM.

Since SEM gives rise to leaves and ultimately flowers, FM and REM give rise to floral organs, we investigated 149 genes whose homologs regulate flowering time or SAM, leaf, and flower development in other plant species (**Supplementary Data 4**). Firstly, we looked at the flowering time genes with functional studies in strawberry. A key gene regulating flowering time in wild strawberry is *FvTFL1*, whose mutation caused the day-neutral as it encodes a floral repressor in the photoperiod pathway (Iwata et al., 2012; Koskela et al., 2012). *FvTFL1* is predominantly expressed in the shoot apex of short-day *F. vesca* grown under long days as examined by RNA *in situ* (Koskela et al., 2012). *TFL1* expression is promoted by *FvSOC1*, the homolog of *SUPPRESSOR OF OVEREXPRESSION OF CONSTANS1*, which is highly expressed under LD in all tissues except flowers (Mouhu et al., 2013). In contrast, two homologs of *FLOWERING LOCUS T*, the florigen, are present in *F. vesca*, called *FvFT1* and *FvFT2*. *FvFT1* is only expressed in old leaves, considered as the ortholog of *FT*, whereas *FvFT2* is expressed exclusively in flower buds (Koskela et al., 2012). Expression patterns of these genes were experimentally validated in *F. vesca*, providing good quality test of our data. Consistent with these results, *FvSOC1* and *FvTFL1* are highly expressed in the SAM, while *FvFT1* is not expressed in any of the three tissues (**Figure 4A**). In contrast, *FvFT2* is expressed in the REM indicating that it may have a different function from *FvFT1*. *FvFT1* and *FvTFL1* are members of the phosphatidylethanolamine binding protein (PEBP) family (Karlgren et al., 2011). We further identified other PEBP family members in *F. vesca*, and found that *FvATC* and *FvBFT* are also greatly expressed in the SAM, suggesting potential functions in the control of flowering time. Consistently, *ATC* is the antiflorigen expressed in old leaves and transported to the shoot apical meristem in Arabidopsis (Huang et al., 2012). *NsCET1*, the homolog of *ATC* in tobacco (*Nicotiana sylvestris*), is demonstrated to move from leaf to the shoot apex *via* long distance transport of mRNA molecules (Huang et al., 2018). The Arabidopsis *BROTHER OF FT AND TFL1* (*BFT*) has a TFL1-like activity and is expressed in the shoot apical meristem, young leaf and axillary inflorescence meristem (Yoo et al., 2010). These analyses helped identify candidate genes that may participate in flowering time regulation in *F. vesca*.

### Analysis of Genes With Roles in SAM and Leaf Development

Our SAM samples consist of both the shoot apical meristem and a young leaf. Thus we expect to see high expression of the key genes regulating the development of both tissues. First, we checked the expression of SAM genes. Among the 149 genes, *FvTAW1-2* and *FvTAW1-4* are predominantly expressed in the SAM, which are the homologs of *TAWAWA1*, a regulator of meristem in rice (Yoshida et al., 2013). In *F. vesca*, there are two close homologs of *WUS*, *FvWUS1* (FvH4_3g04400/gene30464) and *FvWUS2* (FvH4_1g11910/gene14621) (Hollender et al., 2014), which share 36% and 38% protein sequence identity with WUS, respectively. Although the identity scores are moderate, they both possess the conserved homeobox (HB) domain and the WUS_box and EAR motifs (Hollender et al., 2014). According to the RNA-seq data, *FvWUS1* is barely expressed in the SAM but gradually up-regulated in the FM and REM; in contrast, *FvWUS2* is more highly expressed in the SAM and FM compared to REM (**Figure 4A**). However, the *FvWUS2* transcripts are most abundant in anthers (Hollender et al., 2014). It will be interesting to determine if *FvWUS1*, *FvWUS2*, or both are the orthologs of Arabidopsis *WUS* with critical roles in SAM maintenance. In addition, *FvCLAVATA1*, *2*, *3* are abundantly expressed in all three tissue samples, consistent with their roles in Arabidopsis meristems (**Supplementary Data 4**). The REM in this study was obtained from flowers at stages 6–7, when expression levels of *WUS*, *CLV1*, and *CLV3* start to decline in Arabidopsis FM (Clark et al., 1997; Schoof et al., 2000; Xu et al., 2018). Therefore, the abundant expression of these genes in strawberry REM may suggest that the strawberry REM needs to maintain its meristematic activity for an extended period of time to ensure continued enlargement of the receptacle leading to the production of more carpels.

Additionally, a number of known genes important for leaf development are also highly expressed in the SAM, which contains of young leaves. For instance, some leaf development genes are specifically expressed in the adaxial or abaxial domain of young leaves to establish the adaxial-abaxial polarity. Several strawberry homologs of the adaxial-abaxial genes are abundantly expressed in the SAM including an HD-ZIPIII transcription factor *PHAVOLUTA* (*FvPHV*), abaxially expressed *YABBY*s (*FvYAB2*, *FvYAB5*), and *ASYMMETRIC LEAVES1*, 2 genes (*FvAS1a*, *FvAS1b*, and *FvAS2*) (McConnell et al., 2001; Lin et al., 2003; Xu et al., 2003; Stahle et al., 2009). Given that the SAM tissue we isolated contains young leaf primordia, it is not surprising that important leaf development genes are also identified.

### Expression Patterns of Flower Development Genes

In contrast to SAM, FM and REM do not contain leaf primordia and are producers of floral organs, with REM essentially a late stage FM. Expression patterns of 37 homologs of flower development genes in *F. vesca* were shown (**Figure 4A**), including ABCE genes whose expression were previously characterized using LCM-dissected *F. vesca* tissues (Hollender et al., 2014). Here, we revisited their expression patterns in hand dissected SAM, FM, and REM. Consistently, most of them are highly expressed in the FM and REM (**Figure 4A**). One exception is *FvAP2*, which is more highly expressed in the SAM. Previous study in Arabidopsis showed that *AP2* is involved in the stem cell maintenance in the SAM (Wurschum et al., 2006), hence *FvAP2* might play similar roles in strawberry. Additionally, two floral meristem identity genes, *FvLFYa* and *FvAP1*, are greatly induced in FM (**Figure 4A**), consistent with the expression patterns of their homologs in Arabidopsis (Mandel et al., 1992; Weigel et al., 1992).

Next, we examined candidate floral MADS-box genes that may play important roles in strawberry flower development. Based on the BLAST search for the presence of SRF-TF (PF00319) domain, a total of 84 MADS-box genes were identified in the *F. vesca* genome version4.0.a2 (Li et al., 2019). Among them, 27 genes are expressed at a level greater than 2 TPM in one of the three meristems, including 12 type I MADS-box genes (M-box only) and 15 type II MADS-box genes (M-box and K-box) (**Supplementary Data 4**). Moreover, a number of the MADS-box genes are differentially expressed in the FM compared to SAM (**Figure 4B**). Among the type I genes, the SAM-specific gene *FvAGL42* (FvH4_7g28740) is a homolog of Arabidopsis SOC1-like gene *AGL42*, which promotes Arabidopsis flowering in the shoot apical and axillary meristems through the gibberellins-dependent pathway (Dorca-Fornell et al., 2011). There are several MADS-box genes that are greatly induced in the FM (**Figure 4B**), whose functions await further investigation.

### Expression Patterns of the Hormone Pathway Genes

**Figure 5:**
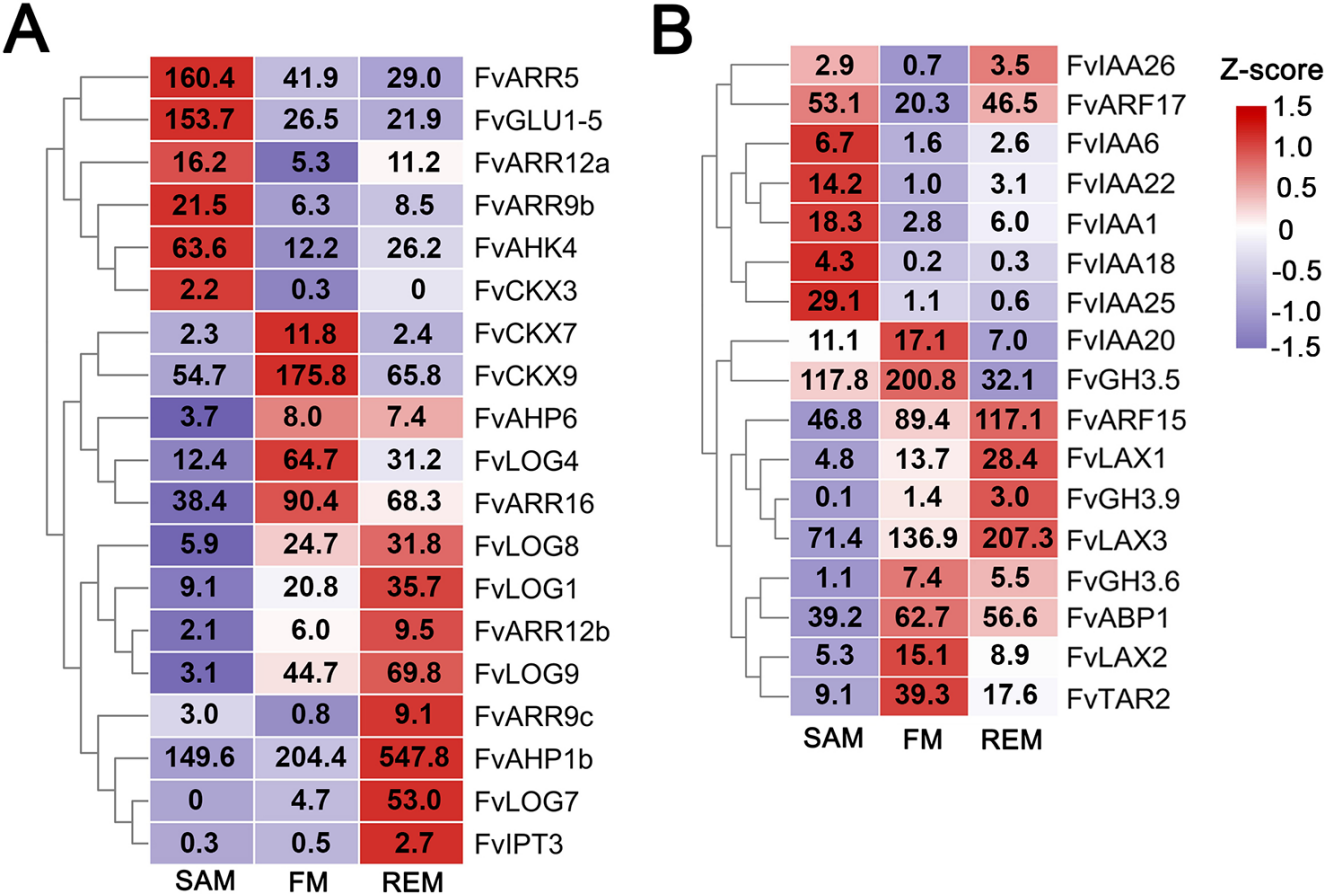
DEGs in the cytokinin and auxin pathways. **(A)** Heatmap showing the expression patterns of differentially expressed cytokinin pathway genes. **(B)** Heatmap showing the expression patterns of differentially expressed auxin pathway genes. Z-score obtained from averaged TPM of three biological replicates was used. The numbers in the heatmap indicate the TPM value of each gene in the tissues.

Cytokinin is a key positive regulatory hormone in meristem maintenance and interacts extensively with the *WUS*/*CLV* module at the biosynthesis, degradation, and signaling processes in the SAM of Arabidopsis (Bartrina et al., 2011; Kieber and Schaller, 2014; Lee et al., 2019). We examined expression patterns of the cytokinin pathway genes in strawberry meristems which were previously identified from *F. vesca* (**Supplementary Data 5**) (Kang et al., 2013). A total of 19 cytokinin pathway genes are differentially expressed among the three meristem tissues (**Figure 5A**). For instance, the biosynthetic genes *FvLOG3* (*LONELY GUY*) and *FvLOG6*, the receptor gene *FvAHK4*, and four downstream *ARR* genes are predominantly expressed in the SAM (**Figure 5A**), indicating a high cytokinin level and active cytokinin signaling in the SAM. In the FM, three *CYTOKININ OXIDASE* (*CKX*) genes are highly expressed, suggesting possibly active degradation of cytokinin. Of note, the cytokinin biosynthesis is greatly enhanced in the REM, supported by the up-regulation of four *LOG* genes, consistent with the rapid growth and enlargement of REM at these stages. These results indicate that cytokinin should also act as a pivotal hormone during the meristem development in strawberry.

Auxin level and signaling in the PZ of the SAM play important roles in the initiation of leaf and flower primordia and in regulating the phyllotaxis (Sassi and Vernoux, 2013). There are totally 17 auxin pathway genes that were differentially expressed in the three meristems. Noticeably, a number of the *AUXIN RESPONSE FACTOR* (*ARF*) and *Aux/IAA* transcription factor family genes are abundantly expressed in the SAM as well as the other two meristems (**Figure 5B**, **Supplementary Data 5**), consistent with the expression study of their Arabidopsis homologs (Vernoux et al., 2011). Especially, *FvARF2* (gene08492/FvH4_2g38760), the homolog of *MONOPTEROS/ARF5* that helps maintain stem cell homeostasis in the SAM (Vidaurre et al., 2007; Luo et al., 2018), is highly expressed in all three meristems. In addition, three *LAX* genes, coding for the auxin influx transporters (Peret et al., 2012), are abundantly expressed in the FM and REM, indicating the importance of auxin transport in their development.

### The Co-Expression Gene Module Associated With Meristem Tissues

**Figure 6:**
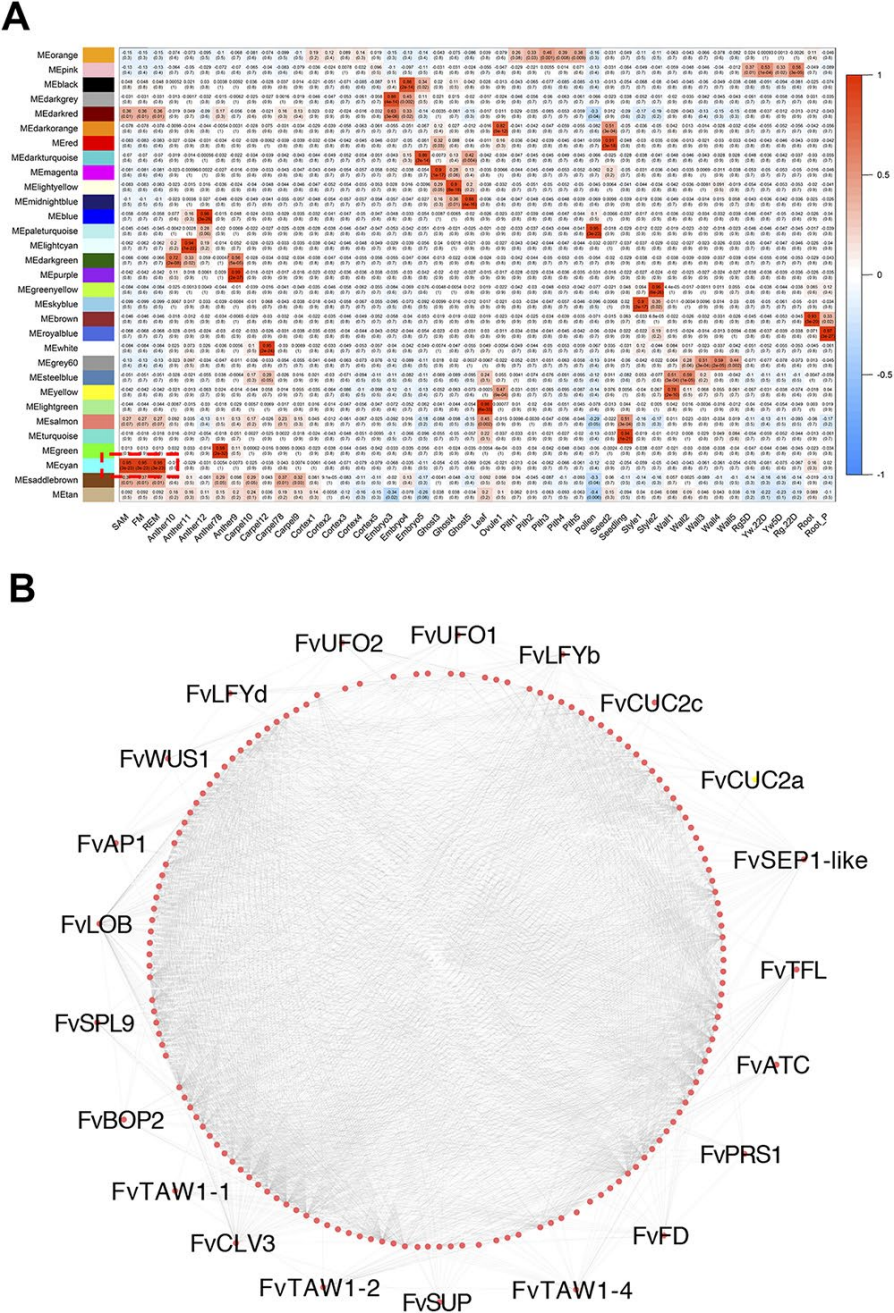
Meristem-associated gene module identified by WGCNA. **(A)** Heatmap showing module-tissue associations of the gene network. Each row corresponds to a module. Each column corresponds to a speciﬁc tissue. The color of each cell at the row-column intersection indicates the eigengene expression value. **(B)** The correlation network of the meristem-associated cyan module. 168 genes with edge weight higher than 0.2 were visualized by Cytoscape. Each dot represents one gene. Lines represent significantly positive correlation between the connected genes.

Taking advantage of the RNA-seq data generated from 43 hand dissected tissues in *F.vesca* (Kang et al., 2013; Hollender et al., 2014; Toljamo et al., 2016; Hawkins et al., 2017) plus three meristem tissues generated here, we constructed a co-expression network using WGCNA (Langfelder and Horvath, 2008). The TPM values of 26,192 genes in these 46 tissues were used for constructing the network, resulting in 31 distinct co-expression modules, each with a different expression profile (**Figure 6A**). Among these modules, the cyan module contains a total of 347 genes that are abundantly expressed in the three meristem tissues as shown by the module eigengene (**Figure 6A**; **Supplementary Data 6**). The gene network of 168 genes with the edge weight higher than 0.2 in the cyan module was visualized by Cytoscape (**Figure 6B**) (Smoot et al., 2010). We found that the cyan module was enriched with transcription factor genes with roles in meristems as mentioned previously (**Figure 4**) including *FvWUS1*, *FvLFYs*, *FvUFOs* and *FvCUC2s*. In addition, some genes constitutively expressed in the three meristem tissues were also identified, such as FvH4_3g43590 that may encode a CLAVATA3/ESR (CLE)-related protein, and the lateral boundary gene *FvLOB* (FvH4_5g05970). The genes in this cyan module might play roles in the regulation of meristem development.

### Detailed Analysis of the *FvWUS1* Expression in Flowers

**Figure 7:**
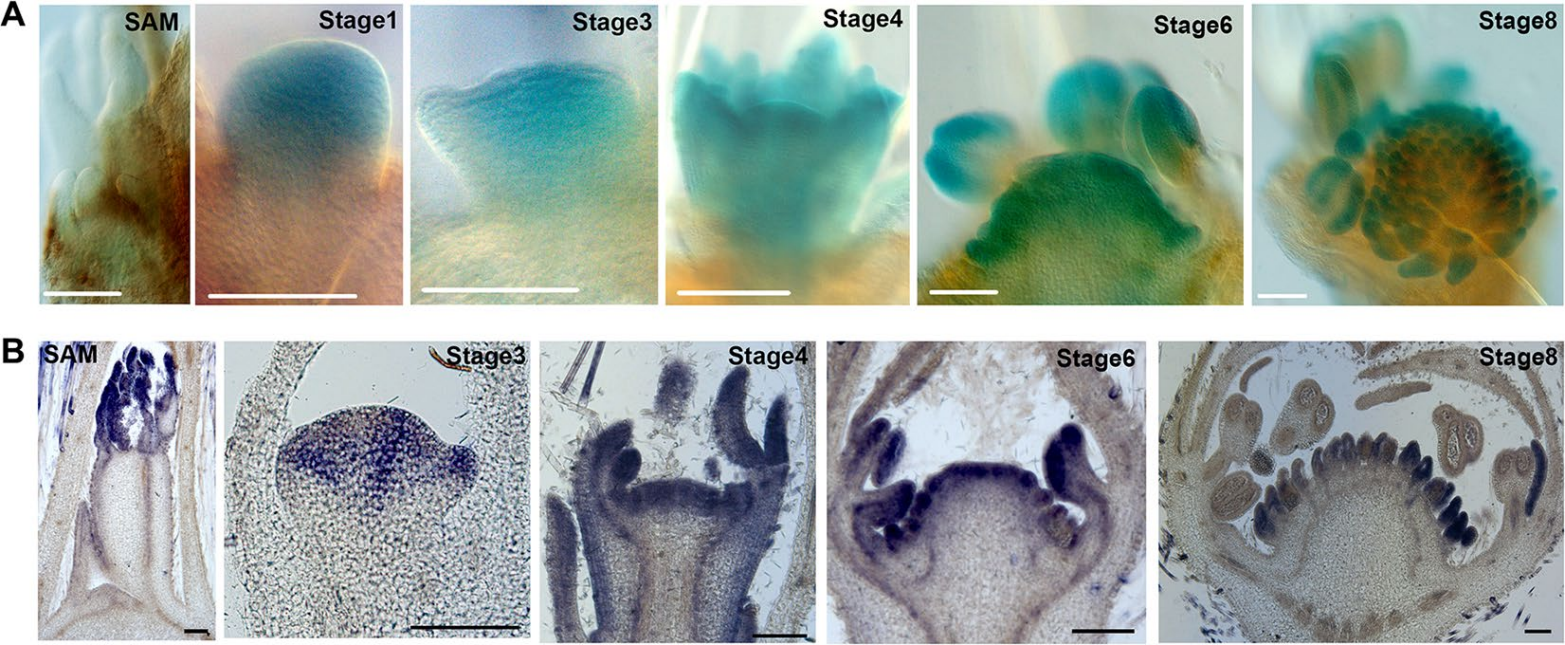
Expression of *FvWUS1* in the SAM and young flower buds of *F. vesca*. **(A)** GUS expression of the *FvWUS1pro::GUS* reporter lines in the SAM and flower buds at stages 1, 3, 4, 6, and 8. The SAM and young flower buds were dissected from five independent T0 transgenic lines. **(B)** Detection of the *FvWUS1* transcripts in the SAM with a young leaf and flower buds at stages 3, 4, 6, and 8. 7 μm longitudinal sections of fixed tissues were used. No signal was detected by the control probe. Scale bars: 100 μm.

According to the RNA-seq data, expression of *FvWUS1* is gradually increased from the SAM to REM (**Figure 4A**), which differs from its homologs in other species. To validate this result, the 2,076 bp promoter of *FvWUS1* upstream of the translational start codon was isolated and used to drive the β-glucuronidase (GUS) reporter. The *FvWUS1pro::GUS* construct was stably transformed into the wild type *F. vesca* variety Hawaii 4. A total of five independent transgenic lines were validated by PCR and then characterized. The SAM and flower buds at stages 1–7 were stained with X-Gluc. In the SAM, no blue color was detected (**Figure 7A**), in agreement with the very low expression level of *FvWUS1* in SAM revealed by the RNA-seq data. In the stages 1–4 flowers, the blue color was detected in the entire FM, but darker in the center region. In stages 6 and 8 flowers, the blue color was present in the young carpels and anthers, which differs dramatically from the restricted expression of *WUS* in the OC (organizing center) of SAM and FM in Arabidopsis (Mayer et al., 1998).

Since the *FvWUS1pro::GUS* transgene could miss cis-regulatory element, RNA *in situ* is more accurate at revealing endogenous *FvWUS* expression. Therefore, *in situ* hybridization was conducted in the SAM and young flower buds. Since *FvWUS1* and *FvWUS2* sequences are highly similar to each other, especially at the 3’ end (**Supplementary Figure S2**), a 163 bp fragment at the 5’ end was used as an *FvWUS1*-specific probe. Consistent with the result of *FvWUS1pro::GUS* reporter expression, *FvWUS1* mRNA is detected in the young FM, at the apex of young receptacle, and carpel and stamen; it is not detected in SAM (**Figure 7B**). Therefore, the expression of *FvWUS1* is distinct from *WUS* expression in Arabidopsis and cucumber (Mayer et al., 1998; Zhao et al., 2018). However, the *WUS* homologs don’t necessarily have exactly the same expression patterns and functions among different species, such as in rice and maize (Nardmann and Werr, 2006; Suzuki et al., 2019). These results confirmed that *FvWUS1* is highly expressed in flower buds at early stages, raising a possibility that *FvWUS1* might play a critical role in regulating the number of carpels and ultimately the receptacle fruit size.

## Conclusion

In this work, we profiled genome-wide transcriptomic landscape of three different tissues, SAM, FM and REM in the diploid strawberry *F. vesca* by Illumina RNA-seq. Detailed analysis was performed on the DEGs derived from pairwise comparisons with a focus on homologs of the crucial transcription factor and hormone pathway genes with roles in meristem maintenance, meristem identity, and meristem differentiation. We found that flower and meristem genes, characterized previously in other species, exhibited both conserved and distinctive expression patterns in strawberry. A novel finding is that *FvWUS1*, a homolog of Arabidopsis *WUS*, is broadly expressed in young flower meristem, indicating its potential function in floral organ initiation and development. These data provides valuable resources for future functional studies of genes with roles in meristem maintenance and differentiation in strawberry.

## Data Availability Statement

The datasets generated for this study can be found in the Sequence Read Archive (SRA) at NCBI (http://www.ncbi.nlm.nih.gov/sra) under the accession number SRP115444.

## Author Contributions

CK and ZL conceived and supervised this study. YL analyzed the data. JF, LC, CD, and QG performed the experiments. CK and YL wrote the manuscript. ZL revised the manuscript. All authors read and approved the final manuscript.

## Funding

This work was supported by the National Key Research and Development Program of China (2018YFD1000102) and National Natural Science Foundation of China (31572098, 31772274, and 31822044).

## Conflict of Interest

The authors declare that the research was conducted in the absence of any commercial or financial relationships that could be construed as a potential conflict of interest.
