# Supplementary material for: Gene Expression Profiling of the Shoot Meristematic Tissues in Woodland Strawberry Fragaria vesca
Source: Front Plant Sci. 2019 Dec 13;10:1624. doi: 10.3389/fpls.2019.01624 (PMC6923813; doi:10.3389/fpls.2019.01624)
Supplement: Supplementary file 7 [file DataSheet_7.docx]

**
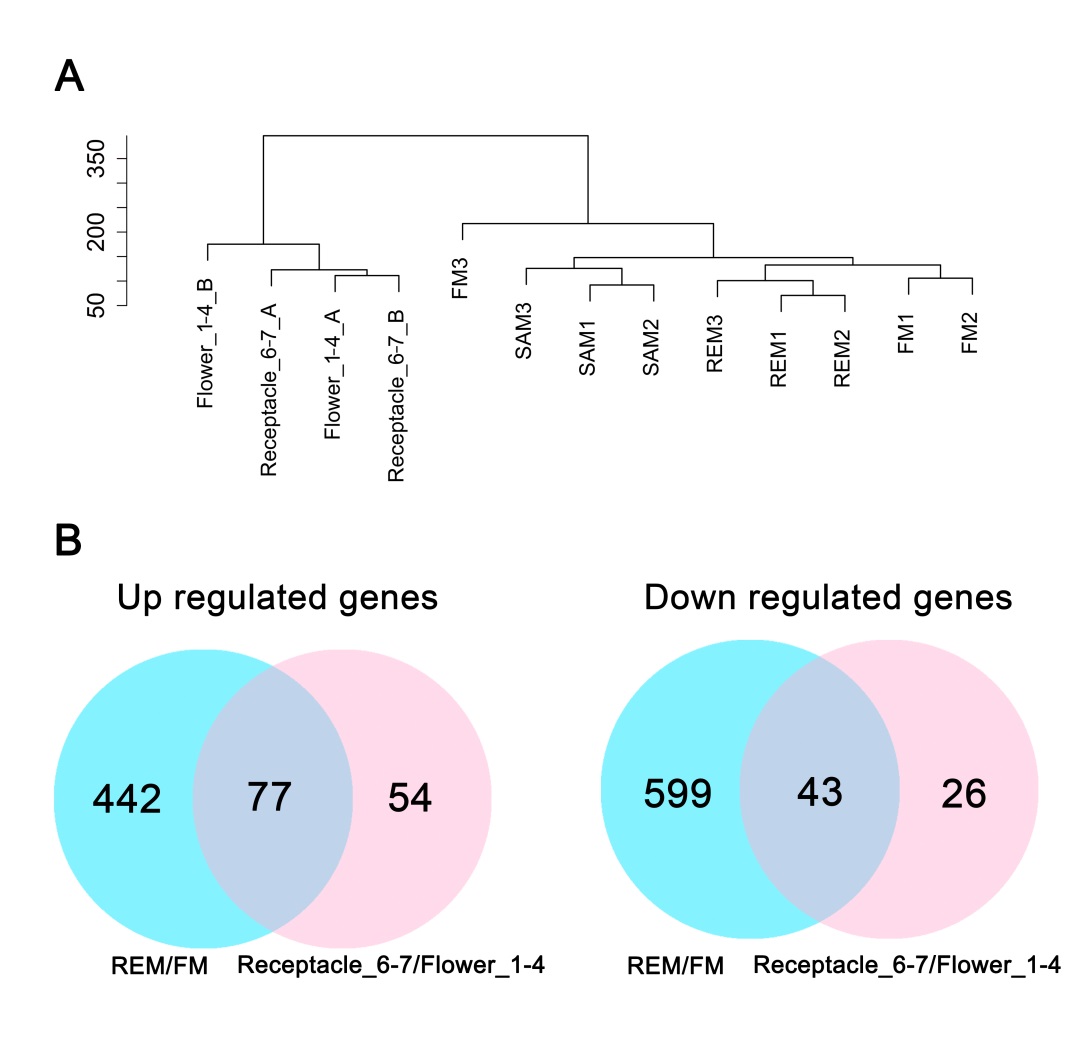
**

**Figure S1. Comparison of the hand-dissected and LCM samples.**

(A) Cluster dendrogram showing global relationships between biological replicates and among different hand-dissected (SAM, FM, and REM) and LCM (Flower_1-4, Receptacle_6-7) tissues. The y axis measures the degree of variance. (B) Venn diagram showing the common and unique differentially expressed genes in the pairwise comparisons (fold change >2, padj < 0.05). Blue circle indicates the comparison between FM and REM. Pink circle indicates the comparison between Flower_1-4 and Receptacle_6-7.


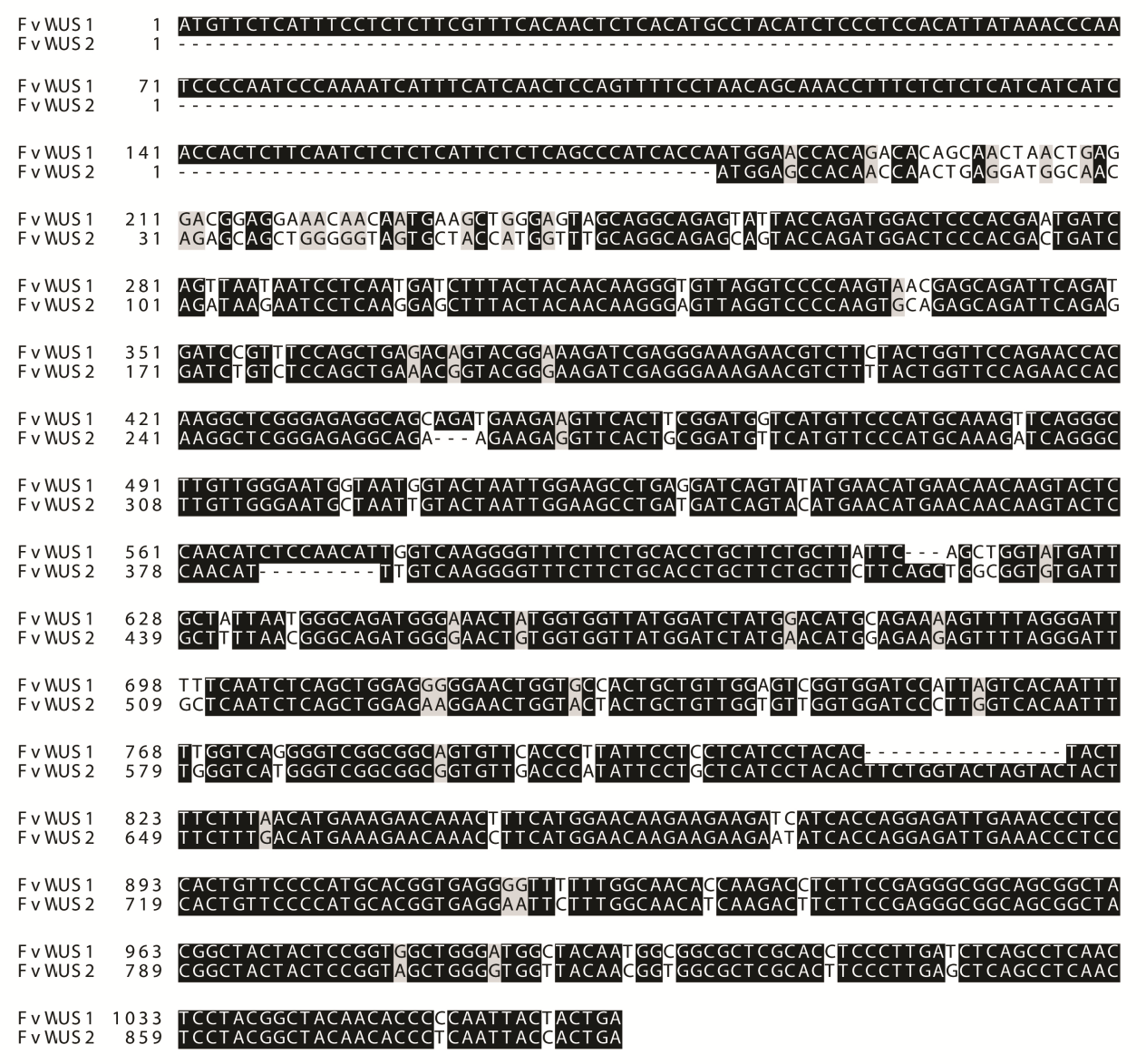


**Figure S2. DNA sequence alignment of *FvWUS1* and *FvWUS2*.**

**Table S1. Summary of RNA-seq read statistics**

| **Sample**ID | **Raw reads** | **Uniquely mapped reads** | **% uniquely mapped** | **No. of expressed**  **genes** |
| --- | --- | --- | --- | --- |
| **SAM1** | 31,543,880 | 27,066,670 | 85.81% | 16,441 |
| **SAM2** | 33,609,145 | 29,761,069 | 88.55% | 16,507 |
| **SAM3** | 34,507,321 | 31,747,748 | 92.00% | 16,384 |
| **FM1** | 30,095,311 | 25,997,151 | 86.38% | 16,254 |
| **FM2** | 33,984,357 | 30,408,084 | 89.48% | 16,694 |
| **FM3** | 32,452,824 | 28,516,414 | 87.87% | 15,106 |
| **REM1** | 32,562,543 | 29,051,173 | 89.22% | 16,708 |
| **REM2** | 33,975,817 | 29,213,020 | 85.98% | 16,547 |
| **REM3** | 29,670,542 | 24,940,442 | 84.06% | 16,456 |
| **Flower_1-4_A** | 27,738,334 | 14,093,392 | 50.81% | / |
| **Flower_1-4_B** | 59,525,934 | 42,052,062 | 70.64% | / |
| **Receptacle_6-7_A** | 15,498,772 | 5,827,568 | 37.60% | / |
| **Receptacle_6-7_B** | 28,074,375 | 13,772,911 | 49.06% | / |
